# Integration of Er^3+^ Emitters in Silicon-on-Insulator Nanodisk Metasurface

**DOI:** 10.3390/nano15191499

**Published:** 2025-10-01

**Authors:** Joshua Bader, Hamed Arianfard, Vincenzo Ciavolino, Mohammed Ashahar Ahamad, Faraz A. Inam, Shin-ichiro Sato, Stefania Castelletto

**Affiliations:** 1 School of Engineering, RMIT University, Melbourne, VIC 3000, Australia; s3904402@student.rmit.edu.au (J.B.); hamed.arianfard@rmit.edu.au (H.A.); s4068868@student.rmit.edu.au (V.C.); 2 Quantum Photonics Laboratory, Centre for Quantum Computation and Communication Technology, School of Engineering, RMIT University, Melbourne, VIC 3000, Australia; 3 Department of Physics, Aligarh Muslim University, Aligarh, Uttar Pradesh 202002, India; ashaharamu2014@gmail.com (M.A.A.); faraz.inam.phy@amu.ac.in (F.A.I.); 4 Quantum Materials and Applications Research Center (QUARC), National Institutes for Quantum Science and Technology (QST), Takasaki, Gunma 370-1292, Japan; sato.shinichiro2@qst.go.jp

**Keywords:** rare-earth erbium ions, asymmetric metasurfaces, photoluminescence, confocal microscopy, photoluminescence excitation spectroscopy, ion implantation, silicon-on-insulator platform

## Abstract

Erbium (${\mathrm{Er}}^{3+}$) emitters are relevant for optical applications due to their narrow emission line directly in the telecom C-band due to the ^4^I_13/2_ → ^4^I_15/2_ transition at 1.54 μm. Additionally, they are promising candidates for future quantum technologies when embedded in thin film silicon-on-insulator (SOI) to achieve fabrication scalability and CMOS compatibility. In this paper we integrate Er^3+^ emitters in SOI metasurfaces made of closely spaced arrays of nanodisks, to study their spontaneous emission via room and cryogenic temperature confocal microscopy, off-resonance and in-resonance photoluminescence excitation at room temperature and time-resolved spectroscopy. This work demonstrates the possibility to adopt CMOS-compatible and fabrication-scalable metasurfaces for controlling and improving the collection efficiency of the spontaneous emission from the Er^3+^ transition in SOI and that they could be adopted in similar technologically advanced materials.

## 1. Introduction

Rare-earth ions in various crystals have been recognized in spectroscopy since the mid-nineteenth century, and from the material science observation domain, it is now possible to deploy their properties in optical technologies, such as lasers, displays, fiber optical communication [1,2,3], integrated photonics [4,5] and more recently in quantum technologies [6,7]. The key appeal of rare-earth ions, from single ions [8,9] right through to large ensembles [10], is the high optical and spin coherence with narrow 4f-4f optical transition homogeneous linewidths at liquid He temperatures, as narrow as 73 Hz or coherence lifetime T_2_ > 4 ms, in Er^3+^:Y_2_SiO_5_ single crystals [11,12], in Er^3+^ in Y_2_O_3_ [9,13], in Si [8,14] and implanted in Si waveguides and photonic cavities [15,16].

In particular, Er^3+^ in silicon-on-insulator (SOI) is a promising material platform as it is a complementary metal–oxide–semiconductor (CMOS)-compatible material and leverages well-established nanofabrication methods to enable scalable integration of quantum networks and quantum memories [17]. One of the key limitations of Er^3+^ in Si and SOI is its very low emission rate, arising from the long optical lifetime (in the order of μs or ms), low quantum efficiency (a few percent), and the high refractive index of the material, which limits photon extraction. Therefore, surface nanostructuring is desirable to enhance the collection efficiency of the Er^3+^ optical transition at 1534 nm.

The use of all high-index dielectric Mie-resonant metamaterials based on Si has been suggested as a viable platform for enhancing the fluorescent emission [18]. Eu^3+^, another trivalent lanthanide ion emitting in the visible range, has shown a maximum absolute averaged fluorescence emission enhancement by a factor of 6.5 by using dielectric metasurfaces composed of Mie-resonant silicon nanocylinders [19].

In this work, we focus on using facile fabrication approaches to achieve scalability of the metasurfaces, by direct fabrication within SOI. In order to achieve maximum overlap between introduced emitter and modes provided by the metasurface, we introduce the Er^3+^ (combined with oxygen co-doping in a 1:10 ratio) via ion implantation at the center of the thin film Si layer, to study the generated fluorescence enhancement properties.

We report the successful integration of ensemble Er^3+^ defects in SOI metasurfaces by means of studying the Er^3+^ photoluminescence (PL) at room temperature (RT) and cryogenic temperature, the PL time-resolved lifetime measurements, the absorption and emission PL polarization as well as room temperature photoluminescence excitation (PLE) properties.

By using confocal microscopy and time-resolved PL at RT, we observed an average maximum experimental PL enhancement of a factor of 5 around the 1535 ± 3 nm transition. The previous implantation of Er in SOI nanopillars provided a fluorescence enhancement of a factor of 2 [20], while a factor of 1.4 enhancement was achieved in erbium-infused silicon nanocones on silica [21], providing an improvement using SOI metasurfaces.

We measured the PL optical lifetime of the Er^3+^ transition in the metasurfaces and in the unpatterned SOI, with a maximum lifetime reduction of 9.6%. When on-resonance excitation (PLE) is considered, we observed a 2.9 photoluminescence enhancement as well as a lifetime reduction of a factor of 2 for an observable Er^3+^ transition at 1534.79 nm. This work paves the way for the utilization of scalable SOI metasurfaces to achieve Er^3+^ emission enhancement through fine-tuning of the metasurfaces’ design.

## 2. Materials and Methods

### 2.1. Er^3+^ Implantation

The ion implantation, was performed using a 400 kV ion implanter at the Takasaki Institute for Advanced Quantum Science, QST. The implantation of 350 keV Er ions at a fluence of $4.0\times 10^{12}\;{\mathrm{cm}}^{-2}$, followed by the implantation of 50 keV O ions at a fluence of $3.0\times 10^{13}\;{\mathrm{cm}}^{-2}$ was performed at a normal angle at room temperature. The ion beams were raster scanned to uniformly implant these ions into the sample. We simulated the Er- and O-ion concentration depth profiles with the stopping and range of ions in matter (SRIM) software package [22], and the implantation energies were chosen to achieve maximal overlap with the metasurfaces. The implanted Er ions were not isotopically separated.

### 2.2. Dielectric Metasurface Fabrication

**Figure 1:**
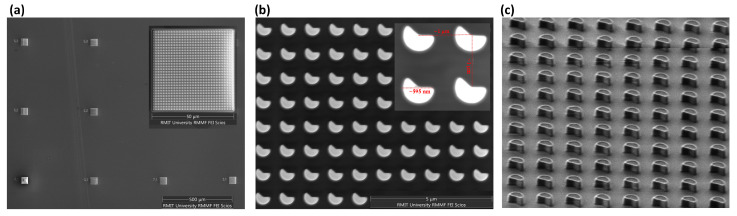
(**a**) Top-view SEM image of the fabricated device showing an array of 50 × 50 μm^2^ square metasurface elements. The inset provides a zoomed-in view of one of the square metasurface elements, revealing its detailed structure. (**b**) Higher magnification SEM view of (**a**), highlighting the symmetry-breaking nanodisk array forming the metasurface. The inset further illustrates the shape and dimensions of the individual symmetry-breaking nanodisks. (**c**) Cross-sectional FIB image of the fabricated nanodisk array, captured at a 52° tilt to reveal the height and structural arrangement of the asymmetric nanodisks.

The metasurfaces were fabricated on an implanted SOI sample with a 260 nm thick top silicon layer (type P/B, orientation: <100>, resistivity 14–22 ohm·cm) and a 2 μm buried oxide (BOX) layer with a nanodisk diameter of 595 nm, pitch of 1 μm and sector cut-angle of 138 ± 2°, based on a previous design methodology predicting theoretical Mie-resonant scattering modes’ collective behavior [23,24]. The fabrication process largely adheres to standard CMOS techniques, except for the patterning step, which was carried out using electron-beam lithography (EBL). Specifically, a Vistec EBPG 5200 electron-beam lithography system was used to define the device pattern on a positive photoresist (ZEP520A). The patterned resist was then transferred to the top silicon layer through inductively coupled plasma (ICP)-reactive ion etching (RIE), utilizing SF_6_ and C_4_F_8_ as the etching gases. Figure 1a shows a top-view scanning electron microscopy (SEM) image of the fabricated device, which consists of an array of square metasurface elements, each measuring 50 × 50 μm^2^. A higher magnification SEM image of a single metasurface element (Figure 1b) reveals the periodic nanodisk array forming the metasurface. The inset further highlights the precise geometry of the nanodisks, confirming their well-defined shape and uniformity. Figure 1c presents a cross-sectional focused ion beam (FIB) image of the fabricated nanodisk array, captured at a 52° tilt to reveal the vertical profile and spatial arrangement of the nanodisks.

### 2.3. Metasurfaces Annealing

In order to optically activate the ${\mathrm{Er}}^{3+}$ defect, thermal annealing was carried out after the metasurfaces’ fabrication at 500 °C [25] and 700 °C [8] for 1 min and 10 min in a $N_{2}$ atmosphere, respectively. Here, we consider both mentioned annealing conditions to further study the impact on the properties of the metasurface. For this, the utilized furnace was heated to each individual target temperature for 1 h under a $N_{2}$ atmosphere while the sample was placed in a glass crucible that connected to the furnace, also under a $N_{2}$ environment. The $N_{2}$ atmosphere was provided with a constant gas flow rate of 2 L/min. Once the initial purge and preheating were completed, the sample was pushed slowly into the hot furnace and annealed for the required time. This technique resulted in steep heating ramps for both conditions. Subsequent cooldown rates were configured to 10 °C/min.

### 2.4. Photoluminescence Spectroscopy

The spectroscopy investigation was carried out using a custom-built confocal microscope with a Thorlabs 976-P300 continuous-wave laser diode for excitation, separating the PL from the laser by a 980 nm longpass dichroic mirror. An Olympus LC Plan 0.65 NA 50× (for the low-temperature investigation) or a 0.85 NA 100× dry objective (for RT investigation) were utilized to direct the excitation laser onto the investigated samples and collect back the fluorescence.

Furthermore, a full polarizer (FP) and a λ/2 waveplate (HWP), at 976 nm, were present to control the excitation polarization during this study and set to achieve maximum PL. A Montana cryostation operating with a closed-loop helium cycle was employed to study the low-temperature properties of the ${\mathrm{Er}}^{3+}$ defect. An ID Quantique InGaAs avalanche photodiode (APD) as well as a Princeton Instruments ${\mathrm{LN}}_{2}$-cooled spectrometer were utilized to detect the emitted infrared photons. Details of the experimental setup are provided in Figure S1 of the Supplementary Materials.

### 2.5. Confocal Imaging

We utilized another custom-built confocal microscope to acquire the high-resolution metasurfaces’ images, which was equipped with a 780 nm continuous-wave laser, a 900 nm long pass dichroic mirror (DM) followed by another 900 nm long pass (LP) filter and a 100× objective (0.85 NA). The fluorescence was focused by an achromatic doublet lens into a single mode (SM) fiber that acted as a pinhole. A Single Quantum EOS-810 superconducting nanowire single-photon detector (SNSPD) captured the emitted infrared photons. The sample was positioned on a PI NanoCube piezo stage with a 100 × 100 μm travel range. The confocal for imaging acquisition was controlled through the open-source software suite Qudi [26]. Details of the experimental setup are provided in Figure S2 of the Supplementary Materials.

### 2.6. Time-Resolved Photoluminescence Measurements

A Thorlabs MC1F2 optical beam-chopper driven by an arbitrary waveform generator was implemented into the excitation section of the confocal microscope (Figure S1 of the Supplementary Materials), which modulated the applied 976 nm excitation for the optical lifetime investigation. A 1550 ± 50 nm bandpass (BP) isolated the ${\mathrm{Er}}^{3+}$ emission.

### 2.7. Polarization Measurements

One FP and one HWP were placed into the emission section of the experimental setup (Figure S1 of the Supplementary Materials), tailored to the respective wavelengths. The already present FP, HWP and BP remained within the excitation/emission– section of the experimental setup, respectively, to investigate the polarization of the observed defect.

### 2.8. Photoluminescence Excitation and Time Resolved in Resonance Excitation

PLE spectroscopy was carried out in a free-space configuration at RT. Tunable excitation (Cobrite DX100), ranging from 192.7924 THz to 195.9439 THz (1530 nm and 1555 nm), was modulated (acoustic–optic modulator AA Optoelectronics MT110-A1.5-IR with extinction ratios greater than 45 dB) and then applied via an Olympus 100× Objective (LCPlan N 100× 0.85 NA) in 500 MHz increments with photon events captured subsequently by an SNSPD. During excitation, the bias current from the SNSPD was set to zero and established back to a valid value at a time range that coincidenced with the falling edge of the excitation. The falling edge of the excitation was simultaneously detected and timestamped (ID Quantique ID801), which was seen as a trigger event. Following that, the incoming timestamps of the photon events were recorded. Via post-processing, we removed all timestamped photon events before the falling edge trigger and were able to extract the amount of detected photons for each applied pulse 1 ms after the trigger event. Resonant optical lifetime transients were obtained by first identifying the inhomogeneous resonances within the PLE spectrum via single Gaussian fitments. Following that, the excitation frequency was configured to drive the determined inhomogenous resonance with a subsequent transient acquisition for 25 ms after excitation extinction. Finally, a bi-exponential fit focusing on the decay part of the transient revealed the optical lifetime.

### 2.9. Collection Efficiency Modeling

Electro-dynamical calculations were carried out utilizing a finite element method (FEM)-based Comsol Multiphysics radio-frequency (RF) module. In these calculations, the Er^3+^ emitter was treated as a radiating point dipole and was modeled as an oscillating point current source driven at the dipole’s emission wavelength λ=1535 nm [27,28]. In order to model a periodic array of nanodisk metasurfaces, periodic boundary conditions (PBCs) were applied to the four vertical faces (side boundaries) of the computational domain. On the top and bottom surfaces of the computational domain, second-order scattering/perfectly matched layer (PML) boundary conditions were applied. During calculations, the minimum mesh size was fixed at 1 nm and the maximum at $\frac{\lambda }{7}$. The permittivity values used for Si and ${\mathrm{SiO}}_{2}$ are based on the following reported values [29,30]. The collection efficiency (CE) is calculated as $\mathrm{CE}=P_{\mathrm{collected}}/P_{\mathrm{total}}$, where $P_{\mathrm{collected}}$ is the time-averaged power flowing through the circular collection lens and $P_{\mathrm{total}}$ is the total time-averaged power radiated by the dipole being collected over a closed surface enclosing the dipole source. A circular collection lens of NA=0.7 was placed at a height of 1.5×λ above the central nanodisk of the metasurface. On the Si nanodisk metasurface, in order to avoid the superposition of the dipole’s radiated power on the collection lens arising due to a periodic array of dipoles, the calculation domain comprised of a finite 7×7 array of nanodisks with a single dipole emitter being embedded within the central nanodisk of the 7×7 array with PML/scattering boundary conditions was applied to the side as well as the vertical boundaries of the computational domain [24]. The results from a 7×7 array of disks is expected to closely resemble the practically infinite metasurface while minimizing the computational cost [31].

## 3. Results and Discussions

### 3.1. Photoluminescence Modification

**Figure 2:**
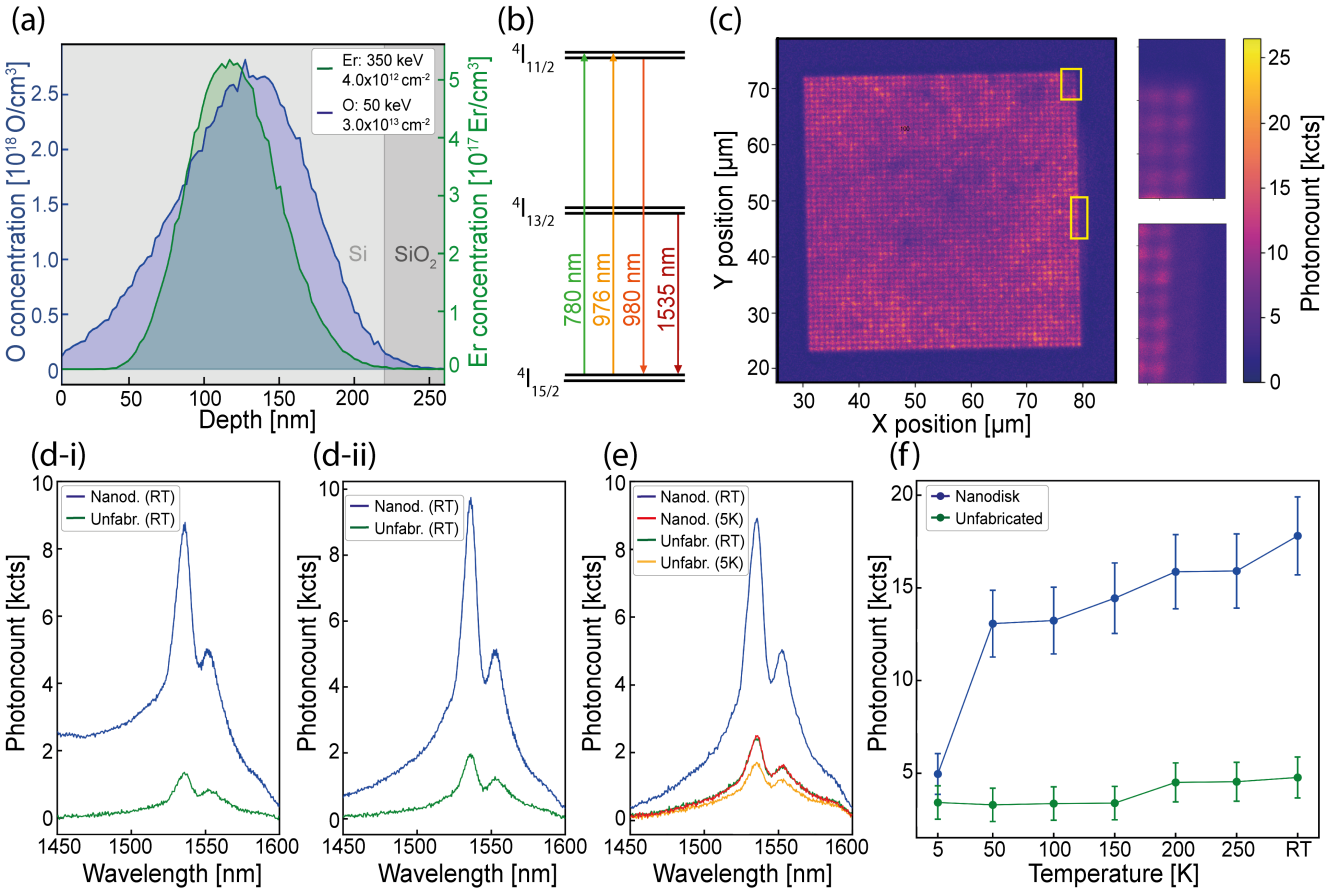
${\mathrm{Er}}^{3+}$ ion implantation and photoluminescence properties: (**a**) Er (green) and O (blue) implantation profile in a 1:10 ratio, focusing on defect generation within the Si layer. (**b**) Energy level diagram illustrating all excited transitions [38]. (**c**) Confocal microscope image scans (yellow boxes are zoom in images shown on the right) illustrating the fabricated nanodisk array with 1 mW excitation at 780 nm in combination with a 900 nm longpass filter (LP). (**d**) Off-resonance PL spectrum obtained for defects within the metasurface or within unfabricated (unfabr.) sections using a NA = 0.85 100× objective, and 2 mW (≈58 kW/cm^2^) of 976 nm excitation considering (**i**) a 500 °C anneal and (**ii**) a 700 °C anneal. (**e**) Off-resonance PL spectrum obtained using a NA = 0.65 50× objective for defects within the metasurface or within unfabricated sections at 5 K and RT, respectively, with 2 mW (≈34 kW/cm^2^) of 976 nm excitation. (**f**) Er^3+^ transition lines’ total photon count rate measured at different temperatures using a NA = 0.65 50× objective with ≈ 34 kW/cm^2^. Data points are determined by integrating the individually obtained Er^3+^ transition line spectra at 1535 nm over 3 nm around the central peak. The illustrated error bars were defined as the square root of the determined integral.

We investigated the SOI metasurfaces with ${\mathrm{Er}}^{3+}$ defects introduced via ion implantation in a 1:10 (Er:O) ratio [32,33,34] during a two-step implantation process, (1) 350 keV $4.0\times 10^{12}$ $\mathrm{Er}/{\mathrm{cm}}^{2}$ and (2) 50 keV $3.0\times 10^{13}$ $O/{\mathrm{cm}}^{2}$, as shown in Figure 2a. The relevant energy level scheme is illustrated in Figure 2b, showing the Er^3+^ ^4^I_13/2_ → ^4^I_15/2_ transition. However, due to off-resonance excitation, we also excited the ^4^I_11/2_ → ^4^I_15/2_ transition.

The samples were imaged utilizing custom confocal microscopes (see Section 2) using 976 nm excitation before and after annealing, and using 780 nm excitation after annealing (as shown in Figure 2c). Details of the experimental setups are provided in the Supplementary Materials. The confocal images revealed bright areas where the nanodisks were fabricated.

We investigated the spectroscopy properties of the Er^3+^ ^4^I_13/2_ → ^4^I_15/2_ transition at 5 K and at RT, for both emitters within the nanodisk array and in the unfabricated sections of the samples. Our findings, illustrated in Figure 2d-i,d-ii, show that the ^4^I_13/2_ → ^4^I_15/2_ transition line is present in both considered annealing steps (500 °C [25] and 700 °C [8]), utilizing a high NA objective at RT. A measured PL enhancement with a factor of 5 can be determined, considering only the ${\mathrm{Er}}^{3+}$ transition line at 1535 ± 3 nm.

By performing lower-temperature spectroscopy, for both cases with a lower NA objective, we observed a strong PL quenching at temperatures below 50 K, while the PL tended to be more stable at higher temperatures, as illustrated in Figure 2e.

Regardless, we still recorded a PL enhancement of 3.7 at cryogenic temperature, considering only the ${\mathrm{Er}}^{3+}$ transition line at 1535 nm. We also observed a weaker shoulder of the emission between 1550 nm and 1552 nm, which can be attributed to Stark splitting effects [35,36]. Further comparisons at other temperatures led to an enhancement factor reduced to 1.45 at 5 K, as illustrated in Figure 2f. The lower enhancement observed at 5 K could be justified by the high oxygen co-doping, inducing different ${\mathrm{Er}}^{3+}$ centers’ emission, as observed in ref. [37] with a similar Er:O ratio. Our work is not focused on the study of the PL temperature effects; rather, it is focused on the PL enhancement effects, and we observed higher enhancement at RT with associated reduced thermal quenching. By applying a larger integration bandwidth, an enhancement reduction was observed, as shown in Figure S3 of the Supplementary Materials.

A higher NA objective at RT provided the highest enhancement (a factor of 5, up to 5.5), recorded for excitation intensities of ≈58 kW/cm^2^ (Figure 2d-ii) and ≈29 kW/cm^2^ (shown in in the Supplementary Materials, Figure S3d), which is consistent with a higher collection efficiency. This indicates that the excitation intensity has a low influence on the enhancement factor; rather, enhancement variation can be seen spatially within the metasurface.

To better resolve the internal structure of the fabricated metasurfaces and spatial effects on PL enhancement, we conducted confocal microscopy scans with an SNSPD, as described in Section 2 (details in Supplementary Materials). Exciting with 1 mW (≈26 kW/cm^2^) off-resonance at 780 nm and using 900 nm longpass filters for the collection of the emitted photons, we observed an enhanced emission in the fabricated area with respect to the background, which is attributed to the ^4^I_11/2_ → ^4^I_15/2_ transition line centered at 980 nm. A scan of 60 μm × 60 μm was acquired with a 500 nm resolution, enabling a detailed visualization of the nanodisk arrays (inset of Figure 2c) as well as a photon emission confinement. From higher-resolution imaging, we observed a different brightness of the Er emission in the metasurfaces, possibly due to a non-uniform activation of the emitters in the whole structure.

### 3.2. Time-Resolved Photoluminescence

**Figure 3:**
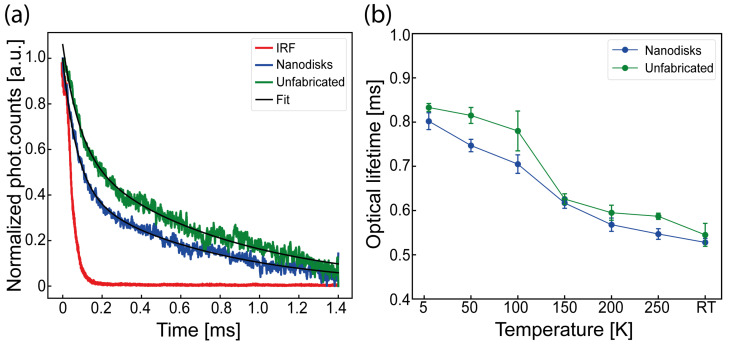
Optical lifetime properties of observed the Er^3+^ transition: (**a**) observed transients from the Er^3+^ defect at 100 K; (**b**) measured optical lifetimes versus temperatures with 976 nm excitation wavelength in combination with a 1550 ± 50 nm bandpass (BP). Lifetimes were measured with the NA = 0.65 50× objective with an intensity of ≈34 kW/cm^2^.

We measured the PL time transients from the ${\mathrm{Er}}^{3+}$ defects to determine the optical lifetime in the metasurface and in unpatterned SOI areas. For this investigation, we modulated the applied optical excitation, synchronized the modulation trigger to the detector output and isolated the relevant emission with a 1550 ± 50 nm bandpass filter (See Section 2 and Supplementary Materials). Here, the instrument response function (IRF) as well as the emission transients obtained from erbium emitters within the nanodisk array and within unfabricated sample sections were fitted with a bi-exponential function (as shown in Figure 3a), as defined in the following equation:

(1) $$\tau_{\mathrm{Fit}}=a\cdot e^{-\frac{t}{\tau_{1}}}+c\cdot e^{-\frac{t}{\tau_{2}}}$$

In Equation (1), a, c, $\tau_{1}$ and $\tau_{2}$ are fitment parameters, where $\tau_{2}$ is the here inferred optical lifetime. Considering this fitting, we determined optical lifetimes of $\tau_{\mathrm{Nanodisks}}\approx 528\pm 9.3$ μs as well as $\tau_{\mathrm{Unfabr}.}\approx 545\pm 26.5$ μs at RT.

We investigated the temperature dependence of the optical lifetime, as shown in Figure 3b, by conducting several measurements over different temperatures, ranging from 5 K to RT, with the maximum lifetime observed at a lower temperature (5 K), for both scenarios, of $\tau_{\mathrm{Nanodisks}}\approx 802\pm 18.5$ μs and $\tau_{\mathrm{Unfabr}.}\approx 833\pm 8.7$ μs, respectively. We observed an overall lifetime variation of 274 μs for emitters embedded in the the nanodisk metasurface and 288 μs for emitters embedded in unfabricated sections of the sample across all temperatures. The lifetime reduction at higher temperature followed the increased PL emission at the same temperature, as previously observed with high oxygen co-doping [37].

The largest impact of the nanodisk design on the lifetime can be observed at 100 K, with a lifetime reduction of approximately 9.6%, with specific lifetimes of $\tau_{\mathrm{Nanodisks}}\approx 705\pm 21$ μs and $\tau_{\mathrm{Unfabr}.}\approx 780\pm 44$ μs, respectively. The lower PL lifetime (<1–2 ms) here observed for the fabricated sample, compared to the unpatterned SOI, can be attributed to a small variation in the local density of electromagnetic states in the nanodisk array due to surface reflectance.

The spectral reflectance measurement of the metasurface did not, in fact, show any resonance at 1535 nm, based on Mie scattering modes’ coherent superposition (see Figure S4 in the Supplementary Materials), as conversely expected by such nanostructure design based on ref. [23], and no evidence of narrow Fano resonances due to the broken symmetry nanodisks was observed, as conversely reported in refs. [39,40]. This justifies the low decay rate enhancement of the metasurface, as similarly observed in close-spaced pillar arrays in silicon carbide [41]. Nevertheless the close spacing of the nanodisks further increased the collection efficiency (See Section 3.4), possibly due to Mie scattering modes of individual nanodisks, resulting in an overall stronger reflectance of the surface and enhanced light extraction from the nanodisks [42]. It is also to be considered that such Mie scattering nanostructures [23,24] or metasurface emission rate enhancement strongly depends on the ensemble emitters’ dipole orientation, which is difficult to simulate, and they are also likely to differ when weak ensemble emitters are considered compared to a single-photon emitter. As such, careful design should be performed to achieve Purcell’s enhancement, which has not been demonstrated so far considering broken symmetry Mie scattering metasurfaces with embedded quantum dots [40,43]. Finally, it has to be noted that the simulation results presented in refs. [23,24] are based on idealized material parameters and nanostructure geometries, while sensitivity studies of deviations in structural dimensions may explain differences between simulated and measured performance, with reduced Q-factors and resonance shifts in experimental implementations.

### 3.3. Metasurface Absorption and Emission Dipole Polarization

**Figure 4:**
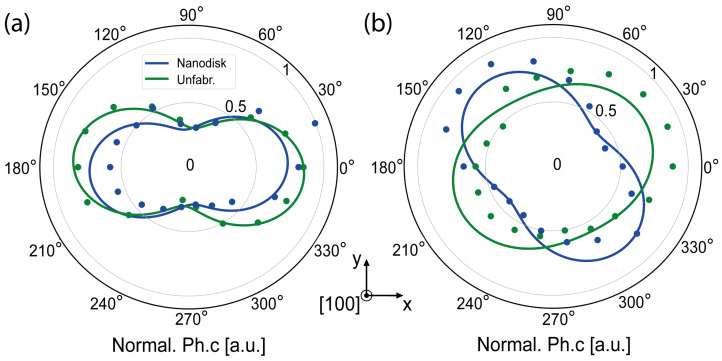
Polarization properties of observed Er^3+^ defects: (**a**) observed photon absorption dipole; (**b**) observed photon emission properties from the ensemble defect.

As illustrated in Figure 4a, the nanodisks’ metasurface had only minor impact on the absorption dipole behavior from the ${\mathrm{Er}}^{3+}$ defects, determined by rotating a 976 nm half-wave plate (HWP) in combination with a full polarizer (FP) with the excitation beam, as shown in the experimental setup of Figure S1 in the Supplementary Materials. We performed a fitting of the measured data based on

(2) $$I=a+b\cdot \sin^{2}\left(\theta_{\mathrm{HWP}}+\phi \right),$$

with a, b and ϕ as fit parameters [44]. Polarization visibilities of $\eta_{\mathrm{Abs}.,\;\mathrm{Nanodisk}}$ ≈ 53.2% and $\eta_{\mathrm{Abs}.,\;\mathrm{Unfabr}.}$≈ 54.2%, respectively, can be identified, calculated as [44]

(3) $$\eta =\frac{I_{\max}-I_{\min}}{I_{\max}+I_{\min}}.$$

Furthermore, we identified unpolarized dipole behavior when studying the emission properties of ${\mathrm{Er}}^{3+}$ defects within unfabricated sections of the sample by rotating a 1550 nm HWP in combination with a FP while the excitation section was set to its respective maximum, as shown in Figure 4b. During this study, a 1550 ± 50 nm BP isolated the relevant emission. A polarization visibility $\eta_{\mathrm{Em}.,\;\mathrm{Unfabr}.}$ of 30.2% could be identified. Interestingly, a polarization visibility $\eta_{\mathrm{Em}.,\;\mathrm{Nanodisk}}$ of 38.2% could be determined in the opposing studied case. We attribute this 8% emission polarization increase to the effects of the nanodisk array. That aligns with the previous presented findings, where a photoluminescence enhancement was identified, and was caused by an increased alignment of the emission dipole with respect to the [100] axis of the crystal within the presented metasurface.

### 3.4. Room Temperature Photoluminescence Excitation and Resonant Time-Resolved Emission

**Figure 5:**
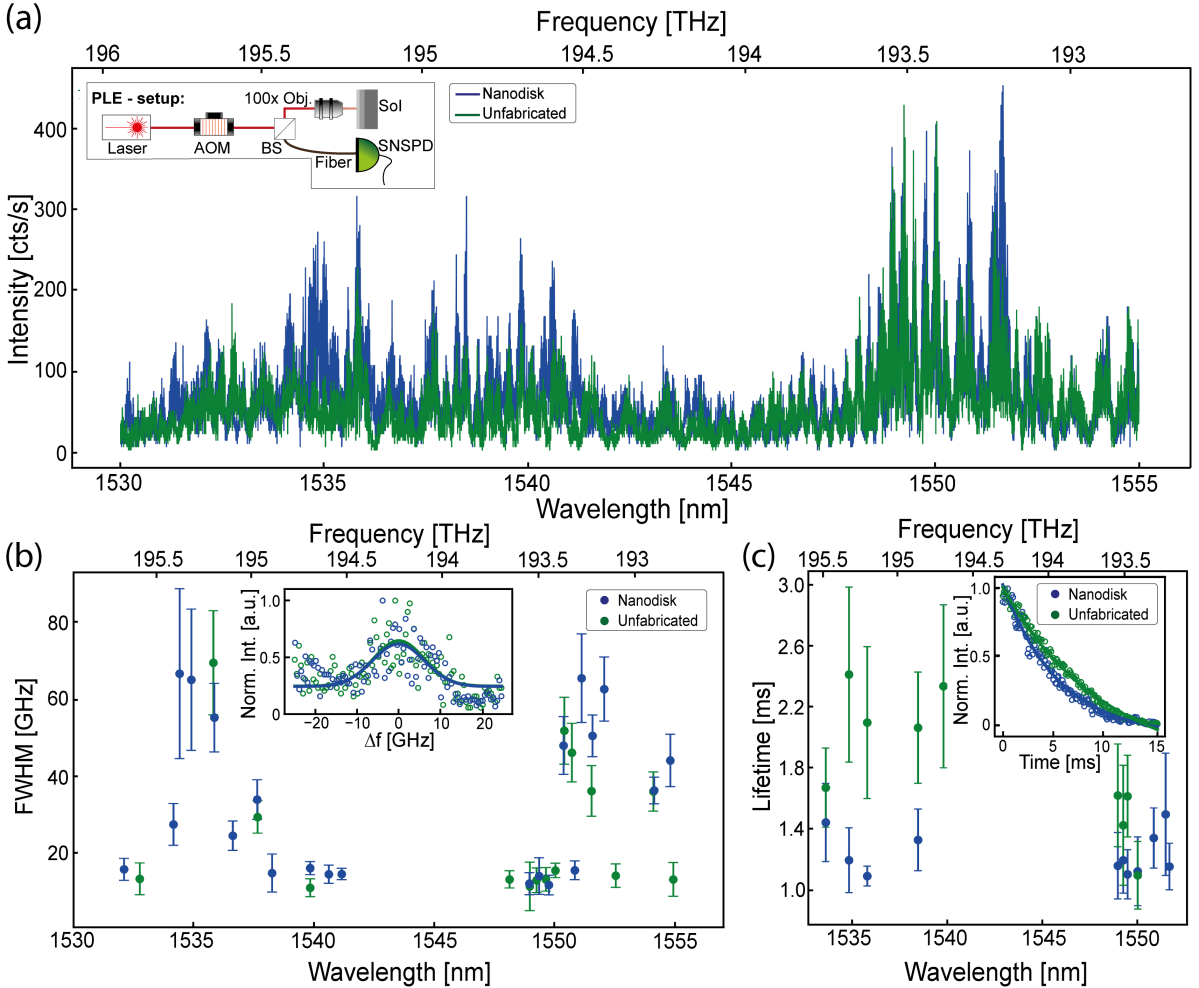
PLE investigation at room temperature: (**a**) obtained photoluminescence excitation spectrum from the Er^3+^-implanted SOI samples in both investigated areas (nanodisks and unfabricated). The inset illustrates the utilized experimental setup schematically; (**b**) exhibited inhomogeneous linewidths for all detected significant Er^3+^ defect resonances in relation to the observed wavelength, where dots represent the linewidth with subsequent fitment uncertainties, illustrated as vertical error bars. The narrowest identified resonances are shown within the inset for both, the nanodisks and the unfabricated area, where a single Gaussian fit (solid lines) is applied to the measured data (dotted); (**c**) lifetime decay overview illustrated with dots in relation to the observed resonance wavelength. Subsequent fitment uncertainties are illustrated as vertical error bars. Normalized measured decay transients obtained over 15 ms (dotted) with subsequently applied bi-exponential fit (solid lines) are shown within the inset from the 1534.84 nm resonance.

By performing photoluminescence excitation (PLE) spectroscopy, we identified individual crystal-field splitting transitions standing in the background of the off-resonance PL spectrum from the generated ${\mathrm{Er}}^{3+}$ defect and investigated the inhomogeneous linewidths and lifetime properties of these transitions at RT. As shown within both PLE spectras in Figure 5a, we can identify two major wavelength regions where distinct ${\mathrm{Er}}^{3+}$ transitions occurred from higher-lying ^4^I_13/2_ fields to the lower-lying ^4^I_15/2_ crystal fields, which also align with the observed broad peaks within the off-resonance PL investigation, as shown in Figure 2d,e: (1) 1534.14 nm until 1541.13 nm and (2) 1548.6 nm until 1551.66 nm. We observe inhomogeneous linewidths with individual full width half maximum (FWHM) values of up to 69.5 GHz ± 13.54 GHz, with the narrowest FWHM values identified at 11.54 GHz ± 2.58 GHz and 10.86 GHz ± 2.32 GHz, as illustrated in Figure 5b. This is significantly broader than previous reports, where inhomogeneous linewidths within the sub-GHz are reported at cryogenic temperatures in SOI [14,15]. Here, the observed broadening could be mostly attributed to the presence of oxygen and the nature of RT investigations.

The observation of wavelength region (2), which is usually only weakly radiative when investigated at cryogenic temperatures, provides new insights into the defect at RT where FWHMs of 11.54 GHz ± 2.58 GHz and 11.03 GHz ± 3.08 GHz were determined for defects embedded into the metasurface or within unfabricated sections, respectively. Furthermore, we observe an individual maximum resonance enhancement factor of 2.9 for a resonance at 1534.79 nm. Interestingly, as opposite, we do not observe significant linewidth modification or photon emission enhancement from resonances situated at 1548.94 nm, 1549.2 nm, 1549.48 nm and 1550.02 nm, which could point to a specific Er site that is not interacting with the metasurface. Individual optical lifetimes from the identified inhomogeneous peaks, as shown Figure 5c, of up to 2.41 ms ± 0.57 ms are observed for defects within unfabricated sections, while the same resonance observed within the metasurface provides a reduced lifetime of 1.19 ms ± 0.21 ms. Not-enhanced resonances in defined section (2) exhibit similar lifetimes of 1.2 ms ± 0.22 ms and 1.42 ms ± 0.39 ms for both scenarios. 

### 3.5. Metasurface Fluorescence Enhancement Model

**Figure 6:**
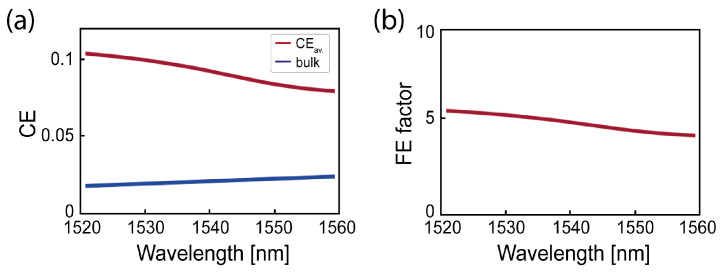
(**a**) The averaged CE spectrum calculated for dipole emission in the nanodisks’ metasurface (red curve) and the unpatterned SOI (blue curve), considering averaged $\left(\phi ,\vec{r}\right)$. (**b**) The FE factor spectrum for ${\mathrm{Er}}^{3+}$ emission in the nanodisks’ metasurface relative to its emission in the unpatterned SOI.

The close spacing of the nanodisks further increased the collection efficiency (CE), resulting in an overall stronger reflectance of the surface at 1535 nm and enhanced light extraction from the nanodisks [42]. We modeled the fabricated metasurface to determine its effect on the photons’ CE, compared to the collection efficiency in the SOI (${\mathrm{CE}}_{\infty }$), used to determine the fluorescence enhancement (FE). The FE can be determined as [45]

(4) $$\mathrm{FE}=\frac{\gamma^{\mathrm{ex}}}{\gamma_{\infty }^{\mathrm{ex}}}\times \frac{\mathrm{CE}}{{\mathrm{CE}}_{\infty }}\times \frac{\mathrm{QE}}{{\mathrm{QE}}_{\infty }},$$

where $\gamma^{\mathrm{ex}}$ is the dipole excitation rate in the metasurface and is scaled with the excitation rate in the unpatterned SOI, $\gamma_{\infty }^{\mathrm{ex}}$. QE and ${\mathrm{QE}}_{\infty }$ are the dipole emission quantum efficiencies in the metasurface and in the SOI, assumed to be the same in the metasurface and the SOI, as the non-radiative decay mechanisms should be maintained. The dipole excitation rate is considered equivalent to the dipole emitter’s total decay rate and can be obtained from the excited state lifetime of the emitter as

(5) $$\frac{\gamma^{\mathrm{ex}}}{\gamma_{\infty }^{\mathrm{ex}}}=\beta ,$$

where β≈ 1.096 is the PL enhancement factor derived from the experimentally total lifetimes in the metasurface and in the unpatterned SOI (Figure 3b at 100 K). The CE and ${\mathrm{CE}}_{\infty }$ for the ${\mathrm{Er}}^{3+}$ emission in the metasurface and the SOI were obtained computationally using a top circular collection lens with a numerical aperture, NA = 0.7, similar to that used in the experimental measurements (Figure 2d). The emitter position *r* and the dipole orientation ϕ were varied following the experimental radiative rate enhancement. The averaged ${\mathrm{CE}}_{\infty }$ for ${\mathrm{Er}}^{3+}$ dipole emission at 1535 nm was calculated to be around 2% in the SOI and CE = 9.5% in the nanodisks’ metasurface. Using the above obtained values for CE and β, the calculated FE enhancement at 1535 nm was determined to be ≈ 5.2 times, as shown in Figure 6, in good agreement with the experimentally observed value of the PL enhancement (Figure 2d).

## 4. Conclusions

To summarize, we demonstrated collection efficiency enhancement of Er^3+^ emitters at 1535 nm using SOI nanodisk metasurfaces. The metasurfaces were fabricated in thin film SOI to demonstrate a CMOS-compatible monolithic integrated erbium emission enhancement material platform. We therefore studied the PL enhancement at RT and cryogenic temperatures, showing a maximum enhancement of a factor of 5 at RT, which is consistent with an ensemble of erbium emitters with low polarization absorption dipoles and a low previous experimental estimate of the internal quantum efficiency of approximately 2.5% [46]. The observed PL enhancement is in agreement with the modeled collection efficiency of such a metasurface of 9.5% compared to 2% of the thin film SOI, and the measured optical lifetime reduction of 9.6%. Considering on-resonance excitation (PLE), we observed longer lifetimes and a lifetime reduction in the metasurface of a factor of 2 for the 1534.79 nm transition, favored by isolating erbium emitters’ radiative decay from non-radiative decay due to the metastable state during off-resonance excitation, while the FWHM of the transition was not reduced by the metasurface due to generally insufficient quality factors. Our results demonstrate that SOI metasurfaces incorporating erbium emitters can be utilized as monolithic integration surfaces to control the spectral and directional properties of the emitters, tailored by the metasurface’s design. Our presented results open interesting opportunities for applications in integrated CMOS-compatible light source technologies in the telecom C-band at RT (e.g., luminescence thermometery [47]), and further enhance the collection efficiency of photons from erbium-doped nanodiodes [48], and could be applied to similar photonic platforms such as silicon carbide on insulator [49]. Future investigations should be directed towards improving the Mie scattering metasurface design to achieve Purcell’s enhancement, which includes considering reduced periods; various radii, with varying asymmetry providing higher Q-factors, as achieved, for example, in non-monolithic quasi-BIC mode slot single nanopillars [50]; and understanding of the effect of closely spaced nanodisk arrays in enhancing collection efficiency.

## Data Availability

The presented data is available upon request from the corresponding author.

## Supplementary Materials

The following supporting information can be downloaded at https://www.mdpi.com/article/10.3390/nano15191499/s1: Figure S1. Experimental setup. Confocal setup for characterizing the ${\mathrm{Er}}^{3+}$ defect fluorescent enhancement; Figure S2. Schematic of the confocal microscopy setup used for imaging the nanodisk array; Figure S3. Further insights into emitters’ properties; Figure S4. Measured reflection spectrum of the metasurface.
