# Supplementary material for: Integration of Er3+ Emitters in Silicon-on-Insulator Nanodisk Metasurface
Source: Nanomaterials (Basel). 2025 Oct 1;15(19):1499. doi: 10.3390/nano15191499 (PMC12525693; doi:10.3390/nano15191499)
Supplement: Supplementary file 1 [file nanomaterials-15-01499-s001.zip › nanomaterials-3900232-supplementary.pdf]

Supplementary Materials: Integration of Er^{3+} Emitters in Silicon-on-Insulator Nanodisk Metasurface

Joshua Bader [1,2], Hamed Arianfard [1,2], Vincenzo Ciavolino [1], Mohammed Ashahar Ahamad [3], Faraz A. Inam [3], Shin-ichiro Sato [4] and Stefania Castelletto [1,*]

1. Additional details from experimental setup

Figure S1 illustrates schematically the confocal setup that was utilized to characterize the Er^{3+} defects' spectral enhancement. In order to capture the optical lifetime of the implanted Er^{3+} defects, a Thorlabs MC1F2 optical beam-chopper (BC) was implemented into the excitation module of the setup and synchronized with the detector output. We added an Edmund Optics 1550±50 nm bandpass (BP) in front of the APD-InGaAs detector in the emission module to isolate the desired emitted signal from the observed Er^{3+} defect. The absorption polarization measurements required a full polarizer (FP1) as well as a rotatable Thorlabs AHWP05M-950 half-wave plate (HWP1) inside the excitation module. Similar components were implemented in the emission module for the subsequent polarizarion study with a Thorlabs WPQSM05-1550 zero-order half-wave plate (HWP2) and an FP2.

Figure S2 illustrates the utilized confocal setup to acquire the images of the metasuface. The excitation was provided by an OZ Optics OZ-3000 fiber-pigtailed ultra-stable laser module with a single mode and polarization-maintaining fiber connector, delivering 1 mW of optical power at a wavelength of 780 nm.

The beam was decoupled from the fiber using a Thorlabs F220FC-780 FC/PC collimator. The power was adjusted using a continuously variable neutral density (ND) filter wheel. An Olympus LCPlan N 100x IR objective with a numerical aperture (NA) of 0.85 focused the beam to a spot approximately 1.1 μm in diameter and applied it to the sample. The sample was mounted on a PI P-611.3 NanoCube piezo stage that enabled precise scanning, offering 100 μm travel in all three axes. The stage was placed on a manual 3D translation stage for coarse positioning and controlled via a PI E-664 digital piezo controller. A Thorlabs DMLP900 longpass dichroic mirror (DM) with a cutoff at 900 nm, followed by a Thorlabs FELH0900 longpass filter (LP), was utilized to isolate the fluorescent signal, expected to be centered around 980 nm. A Thorlabs AC254-100-C achromatic doublet focused the beam into the confocal aperture, which is a single-mode (SM) fiber in this setup.

The collected signal was detected by a Single Quantum EOS-810 superconducting nanowire single-photon detector (SNSPD), featuring a dark count rate below 100 Hz, a dead time under 25 ns, and a timing jitter of less than 50 ps. The detector output consisted of 200 mV pulses with a duration of 50 ns, transmitted via a 50 Ω BNC cable to a custom pulse-level converter circuit, which amplified them to TTL level (∼5 V). The resulting signal was acquired by a National Instruments USB-6343 data acquisition (DAQ) device at a sampling rate of 500 kS/s. The microscope system was controlled using the Qudi software suite [1].

2. Further insights into emitters' properties

We performed spectroscopy and lifetime measurements with a not-annealed sample. We can identify that the Er^{3+} transition line is present in both considered cases, as shown in Fig. S3(a). An enhancement factor of 2.3 can be determined, considered an isolated 1535 nm emission. Lifetime measurements revealed an approximate reduction of 200 μs for defects embedded inside the metasurface. Specific lifetimes of $\tau_{\text{Nanodisks}} \approx 977 \pm 5.5$ μs as well as $\tau_{\text{Unfabr.}} \approx 1.16 \pm 0.026$ ms at RT were determined, as illustrated in Fig. S3(b).

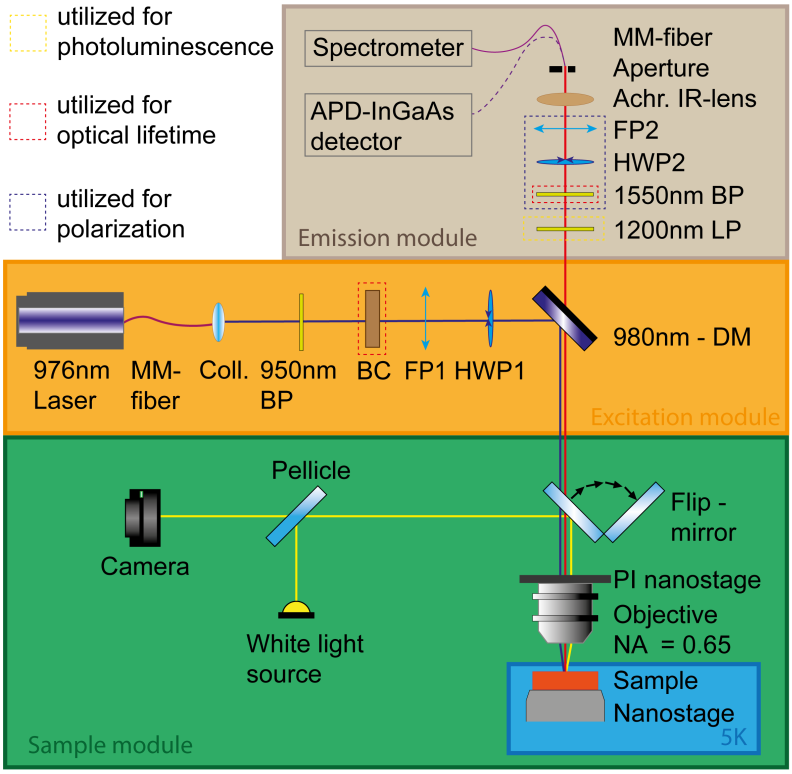

Figure S1. Confocal setup for characterizing the Er-O defect fluorescent enhancement. A fiber-coupled Thorlabs 976-P300 diode laser is focused via a 980 nm dicroic mirror (Semrock Di02-R980-25x36)) onto the sample, utilizing either an Olympus dry objective 100 $\times$, 0.85 NA LCPLN-IR or a 50$\times$ 0.65 NA LCPlan-N objective. This particular excitation passes through a full polarizer and a Thorlabs AHWP05M-950 half-wave plate. The objective is mounted on an XYZ PI P-545.3D8S piezo stage with a 200$\times$200$\times$200 μm travel range. The low-temperature properties were studied with a CryoAdvance Montana Instruments cryostation. The emission was isolated either with a Thorlabs FELH1200 longpass or an Edmund Optics 1550$\pm$50 nm bandpass and subsequently coupled into a multi-mode (MM) fiber via a Thorlabs AC254-100-C achromat. A ID Quantique InGaAs (IDQ230) free-running mode single-photon avalanche photodetector APD (approximately 15% quantum efficiency and $\approx$ 2000 counts/s dark counts) or a Princeton Instruments spectrometer with a Pylon-IR camera LN_2-cooled to -100 °C detected the infrared photons.

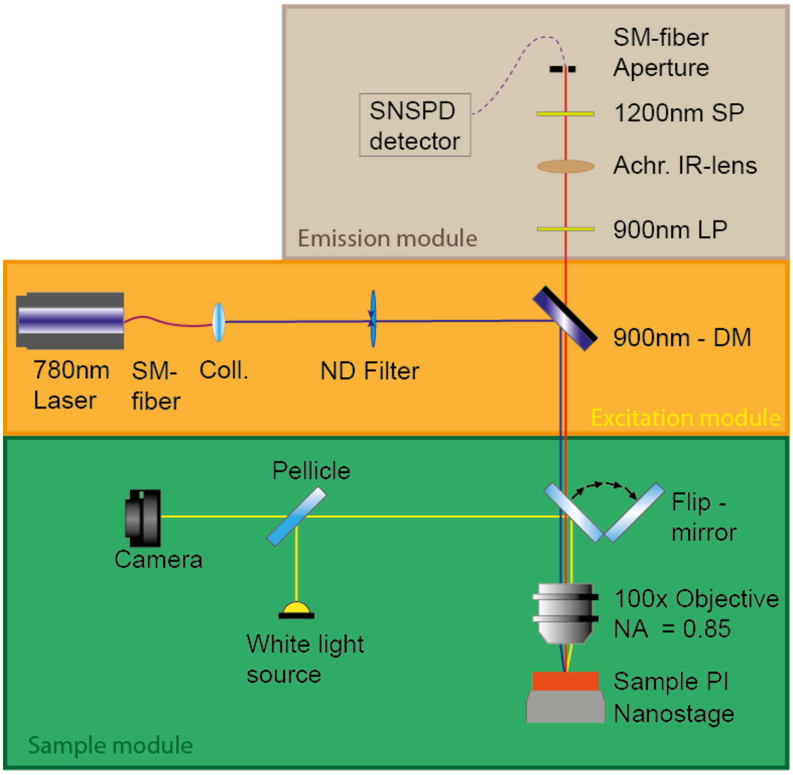

Figure S2. Schematic of the confocal microscopy setup used for imaging the nanodisk array. Excitation at 780 nm is provided by an OZ Optics OZ-3000 fiber-coupled laser and focused onto the sample using an Olympus LCPlan N 100× IR objective (NA 0.85). Scanning is done with a PI P-611.3 NanoCube piezo stage with a 100×100 μm travel range. Fluorescence is collected through the same objective, filtered using a Thorlabs DMLP900 dichroic mirror (DM) and Thorlabs FELH0900 longpass filter (LP), and coupled into a single-mode (SM) fiber. Detection is performed with a Single Quantum EOS-810 superconducting nanowire single-photon detector (SNSPD).

Fig. S3(c) illustrates the impact of different spectral integration bandwidths on the determined enhancement factor, considering the 700 °C annealed sample. Around the 1535 nm central wavelength, with bandwidths of $\pm$ 1, 3, 6 and 9 nm, we identified enhancements of 3.71, 3.65, 3.61 and 3.56 at RT, respectively. Enhancement factors of 1.47, 1.46, 1.43 and 1.41 can be determined at 5K, respectively.

Fig. S3(d) shows the PL enhancement using a 1 mW higher NA objective at RT, showing a similar enhancement of 5.5.

3. Spectral reflectance measurement of the metasurface

The identification of the optical resonance provided by the metasurface was conducted with a commerically available CRAIC Apollo Microspectrophotometer. As shown in Fig. S4, the metasurface provided an reflectance increase, which peaked at 1706.04 nm. The observed secondary peak at 1409.17 nm can also be attributed to the metasurface. From this measurement, a single Gaussian fit reveals a full width half maximum (FWHM) value of 96.69 nm $\pm$ 0.79 nm with a fitted center wavelength of 1703.59 nm, focusing on the primary resonance. This indicates a loaded Q-factor of $\approx$ 17.6. Additionally, a shift from the main optical resonance can be identified compared to the observed Er-transition lines/resonances and explains the low lifetime reduction. Therefore, the experimentally observed PL/PLE enhancement factors and lifetime reductions cannot be linked to the broken symmetry of the metasurface coherent superposition of Mie scattering modes; rather, they can be linked to nanostructure surface effects or individual nanodisks' scattering, increasing, mostly, the collection efficiency.

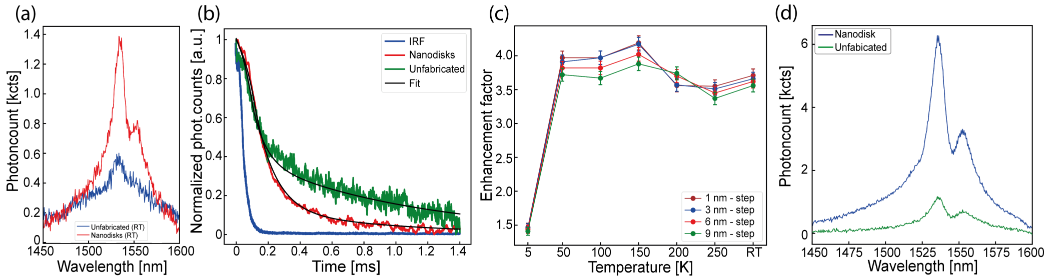

Figure S3. (a) Obtained spectra with a sample in a not-annealed stage obtained with the NA=0.85 100X objective and laser excitation at ≈ 58 kW/cm^2. (b) Optical lifetime transients with a not-annealed sample obtained with an NA=0.85 100X objective and laser excitation at ≈ 58 kW/cm^2. (c) Calculated enhancement factors considering different widths of the Er^{3+} transition line traces with 1535 nm as the center wavelength measured at different temperatures using the NA=0.65 50X objective and laser excitation at ≈ 34 kW/cm^2. (d) PL spectrum from the observed defect utilizing the NA=0.85 100X objective with 976nm excitation at at 1mW (≈ 29 kW/cm^2), indicating an enhancement factor of 5.5.

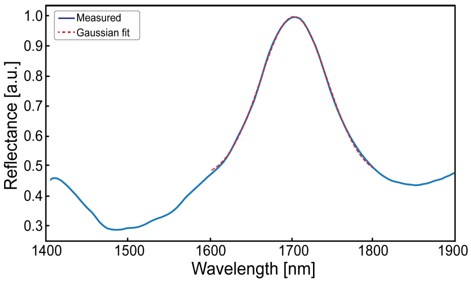

Figure S4. Normalized measured reflectance spectrum from the metasurface (solid blue line) with a subsequently applied Gaussian fit (dashed red line).

1. Binder, J. M.; Stark, A.; Tomek, N.; Scheuer, J.; Frank, F.; Jahnke, K. D.; Müller, C.; Schmitt, S.; Metsch, T.; Unden, T.; Gehring, A.; Huck, A.; Andersen,U. L. ; Rogers,L. J. and Jelezko, F. Qudi: a modular python suite for experiment control and data processing. *arXiv:1611.09146* https://doi.org/10.48550/arXiv.1611.09146.
